# Establishment of the Reference Intervals of Lymphocyte Function in Healthy Adults Based on IFN-γ Secretion Assay upon Phorbol-12-Myristate-13-Acetate/Ionomycin Stimulation

**DOI:** 10.3389/fimmu.2018.00172

**Published:** 2018-02-07

**Authors:** Hongyan Hou, Yu Zhou, Jing Yu, Lie Mao, Munyemana Jean Bosco, Juan Wang, Yanfang Lu, Liyan Mao, Xiaohui Wu, Feng Wang, Ziyong Sun

**Affiliations:** ^1^Department of Laboratory Medicine, Tongji Hospital, Tongji Medical College, Huazhong University of Science and Technology, Wuhan, China

**Keywords:** T cells, NK cells, IFN-γ, reference intervals, whole blood, flow cytometry

## Abstract

The function of lymphocytes is the key to reflect the immune status of hosts. Evaluation of lymphocyte function is a useful tool to monitor the effect of immunosuppressive treatment and predict the prognosis of immune-mediated diseases (e.g., cancer, autoimmune diseases, and infectious diseases). As the lymphocytes have various activities, such as activation, cytotoxicity, and cytokine secretion, it is a challenge to evaluate the function of lymphocytes in clinical practice and the reference intervals (RIs) of lymphocyte function are rarely reported. The present study showed that the secretion of IFN-γ was well correlated with the activation, chemotaxis, and cytotoxicity of CD4^+^, CD8^+^ T cells, and NK cells, which suggests that IFN-γ production can be used as a symbol of lymphocyte function. We therefore created a simple method to detect the function of CD4^+^, CD8^+^ T cells, and NK cells simultaneously according to IFN-γ secretion by using whole blood instead of peripheral blood mononuclear cells. We further established the RIs of lymphocyte function (CD4^+^ T cells: 15.31–34.98%; CD8^+^ T cells: 26.11–66.59%; NK cells: 39.43–70.79%) in healthy adults. This method showed good reproducibility for the evaluation of lymphocyte function. The established RIs were suitable for use in other centers based on the validation data. We also validated the RIs in individuals with different immune status, and the results showed that kidney transplant recipients and infants (0–1 year) had a decreased lymphocyte function, whereas T cells in systemic lupus erythematosus patients exhibited an opposite trend. Overall, we have successfully established the RIs of lymphocyte function in healthy adults in a simple way, which might be of important clinical value in the diagnosis, monitoring, and prognosis of immune-related diseases.

## Introduction

Lymphocytes play critical roles in protecting the host from pathogen invasion, whereas inappropriate activation may contribute to tissue damage. Lymphocyte function analysis could be utilized as an important tool to monitor the immune status of patients and elucidate the pathophysiological mechanisms of immune-mediated diseases. However, the markers which can be used as a symbol of lymphocyte function is unclear due to the lymphocytes have various activities such as activation, cytotoxicity, and cytokine secretion. Therefore, it is a challenge to evaluate the function of lymphocytes in clinical practice conveniently, and the reference intervals (RIs) of lymphocyte function are rarely reported.

The activity of lymphocytes remains stable in healthy individuals over years unless disease occurs. However, newborns or patients with primary immunodeficiencies and late stage of HIV infection have been reported to have decreased T cell and NK cell activity (1–3). The secretion of IFN-γ, TNF-α, and IL-2 by T cells was also significantly decreased in organ transplant recipients receiving immunosuppressive therapy (4). In addition, previous studies have reported that impairment of lymphocyte function is associated with the risk and mortality of cancer (5, 6). In contrast, an overwhelming immune response might contribute to autoimmune diseases, allergic disorders, and graft rejection (7, 8). Thus, evaluation of lymphocyte function has great importance in the diagnosis, monitoring, and prognosis of immune-related diseases.

Several efforts have been taken to define the function of immune cells in the past decades. Traditional methods such as [^3^H]-thymidine incorporation (9) and chromium (^51^Cr)-release test (10) have the hazards of radiation and are not suitable to be used in routine laboratories. Recently, flow cytometry-based assays such as carboxyfluorescein diacetate succinimidyl ester (CFSE) labeling assay with anti-CD3/anti-CD28 stimulation (11) and CD107a degranulation assay after K562 cell line or phorbol-12-myristate-13-acetate (PMA)/ionomycin stimulation are used for analysis of the proliferation and cytotoxicity of lymphocytes (12, 13). Although these methods do not require hazardous materials, they are complicated and the method such as CFSE proliferation assay needs several days to induce the proliferation of lymphocytes. Furthermore, after PMA/ionomycin stimulation, detection of intracellular cytokine production by flow cytometry or evaluation of cytokine profiles by multi-plex technology are also considered as potential methods for monitoring immune function (14, 15). However, whether the production of cytokines can reflect multiple aspects of lymphocyte function has not been fully demonstrated. Therefore, the methods which can simply, rapidly, and accurately determine the function of multiple lymphocyte subsets simultaneously are still needed in clinical practice.

Some studies have reported the reference values of Th1 and Th2 cell function by detecting IL-2 and IFN-γ secretion (16, 17), or obtained the RIs for NK cell activity by using cytotoxicity assay (18). In addition, our previous study has established the RIs of NK cell function through detecting intracellular IFN-γ production and degranulation activity (19). These studies were of great value in understanding the important roles of T cell or NK cell abnormalities in different pathological situations. However, the procedures of these methods are complicated and the established RIs cannot be used to monitor the function of T cells and NK cells simultaneously.

In this study, we created a simple method to detect the function of CD4^+^, CD8^+^ T cells, and NK cells simultaneously by using whole blood after 4 h stimulation. According to the secretion of IFN-γ, we further established the RIs of CD4^+^, CD8^+^ T cell, and NK cell function in healthy adults. The established RIs have practical value based on the validation data in another center and different disease models.

## Materials and Methods

### Study Subjects

This study was carried out in accordance with the recommendations of ethical committee of Tongji hospital, Tongji Medical College, Huazhong University of Science and Technology with written informed consent from all subjects. All subjects gave written informed consent with the Declaration of Helsinki. The protocol was approved by the ethical committee of Tongji hospital, Tongji Medical College, Huazhong University of Science and Technology. Two hundred healthy individuals (92 males and 108 females) aged 20–65 years were recruited from Tongji Hospital, the largest hospital in central region of China. These subjects were determined by interview and physical examination to be healthy and without any clinical symptoms or signs of diseases. Exclusion criteria were as follows: pregnancy, atherosclerosis and vascular disease, cardiopathy, chronic nephropathy, hepatobiliary disease, allergic disease, autoimmune disease, hematological disease, myopathy, burns and muscle trauma, positive for HIV, HBV, HCV, CMV, and syphilis antibodies, and receiving medical treatment. To validate this study, another 100 healthy adults (50 males, 50 females) who met the same inclusion criteria were recruited from Sino-French New City Branch of Tongji Hospital. This study also recruited three groups of individuals with different immune status: (1) 20 kidney transplant recipients (KTR) under immunosuppressive therapy (tacrolimus, azathioprine, and prednisone); (2) 20 infants (0–1 year) without any clinical signs of diseases; and (3) 20 newly diagnosed systemic lupus erythematosus (SLE) patients (according to the revised criteria for SLE of the American College of Rheumatology) before treatment. All participants were Chinese Han nationality.

### Cell Preparation and Activation

Heparinized venous blood was collected from healthy adults. Peripheral blood mononuclear cells (PBMCs) were isolated from whole blood by Ficoll-Hypaque density gradient centrifugation. In order to measure intracellular cytokines, PBMCs (2.5 × 10^5^) were seeded in 96-well plates in 100 µl of medium and stimulated with PMA (50 ng/ml, Sigma-Aldrich) and ionomycin (1 µM, Sigma-Aldrich) for 4 h in the presence of brefeldin A (1 µg/ml,BD Pharmingen). PBMCs were also stimulated with IL-2 (1,000 U/ml, eBioscience), IL-12 (50 ng/ml, eBioscience), anti-CD3 (eBioscience, 10 µg/ml) plus anti-CD28 (eBioscience, 10 µg/ml), or phytohemagglutinin (PHA, 5 mg/ml, Sigma-Aldrich) for 24 h. Brefeldin A was added to the cell culture medium during the last 6 h of incubation. After stimulation, the cells were collected and analyzed by flow cytometry.

### Flow Cytometric Analysis

Cell surface marker staining was performed on unstimulated or stimulated PBMCs. The following monoclonal antibodies were added to the cell suspensions: anti-CD3-FITC, anti-CD3-PerCP/Cy5.5, anti-CD4-FITC, anti-CD4-PerCP/Cy5.5, anti-CD8-PE, anti-CD56-PE, anti-CD56-FITC, anti-HLA-DR-FITC, anti-CCR7-FITC, and anti-NKG2D-FITC (BD Pharmingen). Isotype controls with irrelevant specificities were included as negative controls. All of these cell suspensions were incubated for 30 min on ice. For intracellular staining, the cells were fixed and permeabilized with Fixation/Permeabilization Buffer (BD Biosciences) and stained with anti-IFN-γ-APC, anti-IL-2-PerCP/Cy5.5, anti-TNF-α-FITC, anti-IL-17-PE, anti-IL-21-PE, and anti-TGF-β1-PE (BD Pharmingen) for 30 min in the dark. After washing with the staining buffer, the cell pellets were resuspended in 300 µl cold staining buffer, followed by analysis with FACSCalibur flow cytometer (Becton Dickinson). Data analysis was performed using FlowJo version 9 software (TreeStar).

### CD107a Degranulation Assay

The degranulation assay was tested by the expression of CD107a as described previously (12). Briefly, PBMCs were co-cultured with human leukemic K562 cells for 1 h in the presence of anti-CD107a-FITC antibody (BD Pharmingen) or isotype control. After that, monensin (1 µM, eBioscience) was added to culture medium and incubated for additional 5 h. After culture, cell mixture was stained with anti-CD3-PerCP/Cy5.5, anti-CD4-V450, anti-CD8-APC, and anti-CD56-PE antibodies (BD Pharmingen) and analyzed by FACSCanto flow cytometer (Becton Dickinson).

### Cytotoxicity Assay

The cytotoxicity assay was performed as described previously (20). Briefly, PBMCs were collected as effector cells. K562 cells labeled with 5 µM CFSE (Sigma-Aldrich) were used as target cells. Effector cells and target cells were co-incubated at effector-to-target (E:T) ratio of 10:1 in duplicate wells for 6 h at 37°C with 5% CO_2_. Control tubes including only target cells were analyzed to determine spontaneous cell death. After two washings, 5 µl of propidium iodide (PI, BD Pharmingen) was added to the cell suspensions and the cells were incubated for 15 min on ice in the dark. The cells were then quantified by flow cytometry and dead target cells were indicated as the CFSE^+^ PI^+^ cells.

### Standard Procedures for Whole Blood IFN-γ Secretion Assay

The detection of IFN-γ secretion by CD4^+^, CD8^+^ T cells, and NK cells in whole blood was according to the following procedures: (1) 100 µl of whole blood was diluted with 400 µl of IMDM medium (Gibico-BRL) in 12 × 75 mm polystyrene round-bottom tubes with caps (Falcon 352054, Becton Dickinson). (2) The diluted whole blood was stimulated with Leukocyte Activation Cocktail (BD GolgiPlug™, including 50 ng/ml PMA, 1 µM ionomycin and 1 µg/ml brefeldin A) for 4 h at 37°C with 5% CO_2_. (3) After stimulation, 300 µl of supernatant was aspirated, then five kinds of antibodies (anti-CD45-APC/H7, anti-CD3-FITC, anti-CD4-V450, anti-CD56-PE/Cy7, and anti-CD8-APC) (BD Biosciences) were added to cell suspensions and incubated for 15 min at room temperature. (4) After lysis of erythrocytes, the cell suspensions were fixed and permeabilized with Fixation/Permeabilization Buffer for 20 min at room temperature. (5) After washing, the cell suspensions were stained with anti-IFN-γ-PE (BD Pharmingen) for 20 min at room temperature. (6) After washing, the cell pellets were resuspended in 200 µl PBS and analyzed with FACSCanto flow cytometer (Becton Dickinson).

### Statistical Analysis

Statistical significance between different groups of participants was analyzed using the Mann–Whitney *U*-test. Spearman’s rank correlation test for non-parametric data was employed to analyze the relationship between two factors. The RIs of lymphocyte function were determined by using the 2.5–97.5 percentile nonparametric range (21). The statistical analysis was performed using GraphPad Prism version 5.01 (GraphPad Software, San Diego, CA, USA). Statistical significance was determined as *p* < 0.05 (*p < 0.05, **p < 0.01, ***p < 0.001).

## Results

### The Secretion of IFN-γ Can Reflect the Function of T Cells and NK Cells

**Figure 1:**
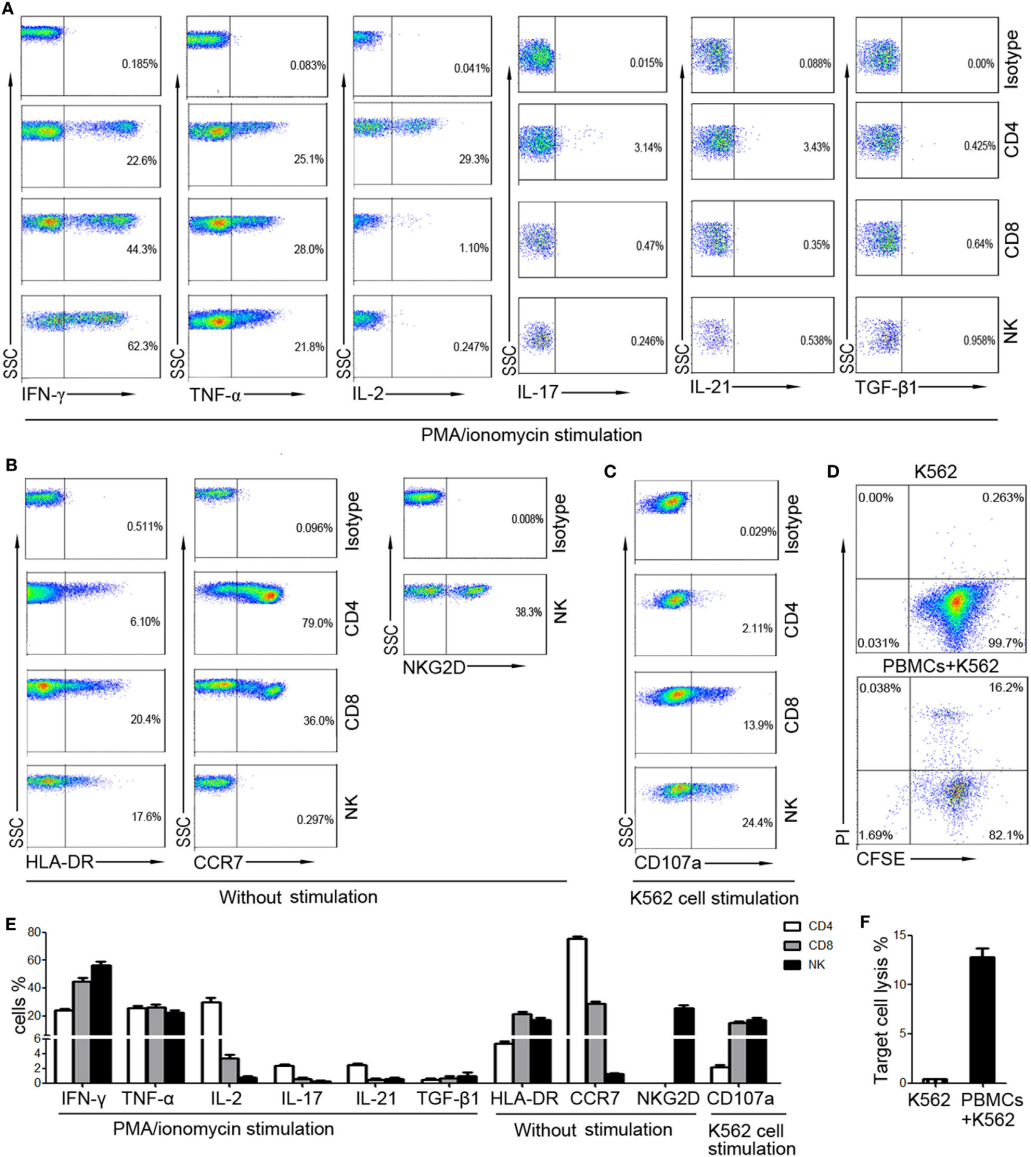
The expression of different markers on CD4^+^, CD8^+^ T cells, and NK cells. Peripheral blood mononuclear cells (PBMCs) were isolated from whole blood of healthy adults (n = 30). **(A)** Isolated PBMCs were incubated with phorbol-12-myristate-13-acetate/ionomycin and the intracellular production of cytokines was analyzed by flow cytometry. Representative FACS plots showing the expression of IFN-γ, TNF-α, IL-2, IL-17, IL-21, and TGF-β1 in CD4^+^, CD8^+^ T cells, and NK cells. **(B)** The expression of HLA-DR and CCR7 on CD4^+^, CD8^+^ T cells, and NK cells and the expression of NKG2D on NK cells were analyzed without stimulation. Representative FACS plots showing the expression of HLA-DR, CCR7, and NKG2D on different subsets of lymphocytes. **(C)** PBMCs incubated with K562 cells were analyzed for the expression of CD107a. Representative FACS plots showing the expression of CD107a on CD4^+^, CD8^+^ T cells, and NK cells. **(D)** PBMCs were incubated with CFSE-labeled K562 cells and the cytotoxicity of PBMCs was analyzed. Representative FACS plots showing the spontaneous lysis of target cells and the lysis of target cells incubated with PBMCs. **(E)** Bar graphs showing the percentages of IFN-γ^+^, TNF-α^+^, IL-2^+^, IL-17^+^, IL-21^+^, TGF-β1^+^, HLA-DR^+^, CCR7^+^, and CD107a^+^ cells in CD4^+^, CD8^+^ T cells, and NK cells, respectively. The percentage of NKG2D^+^ cells in NK cells was also shown. Data are shown as mean ± SEM. **(F)** Bar graphs showing the percentages of lysed target cells in different groups. Data are shown as mean ± SEM.

To screen suitable markers for reflection of lymphocyte function, the cytokine secretion, activation, and cytotoxicity of T cells and NK cells from healthy individuals were investigated. The intracellular cytokines were detected after stimulation with PMA/ionomycin, as the levels of intracellular cytokines were very low without stimulation. Our results showed that high level of IFN-γ and TNF-α can be detected in CD4^+^, CD8^+^ T cells, and NK cells. However, the production of IL-2, IL-17, and IL-21 was mainly detected in CD4^+^ T cells, and the level of TGF-β1 was very low in both T cells and NK cells (Figure 1A). Furthermore, the activation marker (HLA-DR, NKG2D) and chemokine receptor (CCR7) can be detected without stimulation. However, CCR7 was expressed on T cells but not on NK cells, while NKG2D was expressed on NK cells but not on T cells (Figure 1B). We also found that the expression of CD107a on CD8^+^ T cells and NK cells was relatively high, while its expression on CD4^+^ T cells was low (Figure 1C). The percentages of these markers were shown in Figure 1E. The whole cytotoxicity of PBMCs to K562 cells was also detected (Figures 1D,F). Finally, although IFN-γ, TNF-α, and HLA-DR can be detected in both T cells and NK cells, the level of TNF-α and HLA-DR was relatively lower than that of IFN-γ.

**Figure 2:**
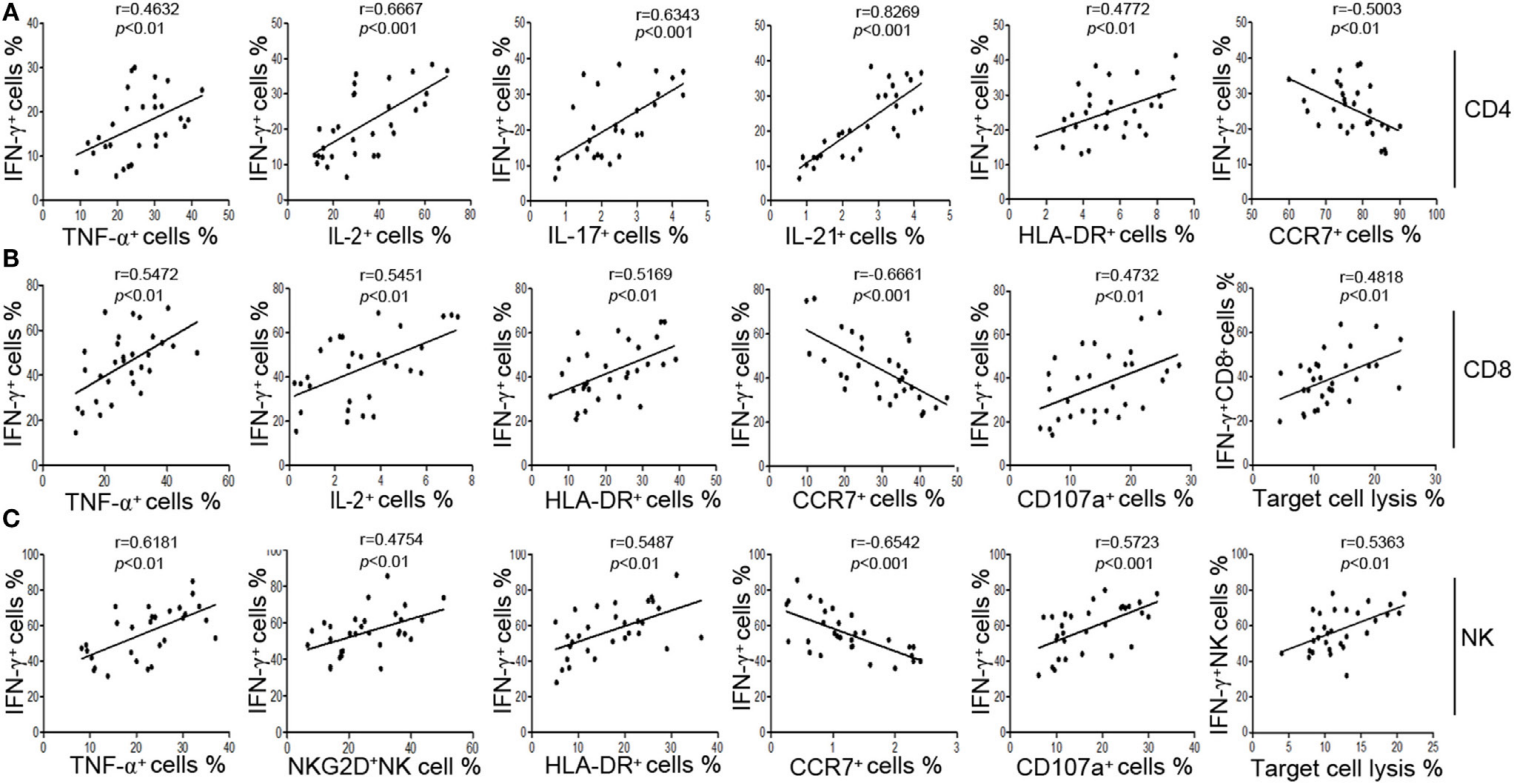
The secretion of IFN-γ can reflect the function of CD4^+^, CD8^+^ T cells, and NK cells. **(A)** Correlation between the percentage of IFN-γ^+^ cells and the percentages of TNF-α^+^, IL-2^+^, IL-17^+^, IL-21^+^, HLA-DR^+^, CCR7^+^ cells in CD4^+^ T cells. **(B)** Correlation between the percentage of IFN-γ^+^ cells and the percentages of TNF-α^+^, IL-2^+^, HLA-DR^+^, CCR7^+^, CD107a^+^ cells in CD8^+^ T cells, and correlation between the percentage of IFN-γ^+^ CD8^+^ T cells and the cytotoxicity of peripheral blood mononuclear cells (PBMCs). **(C)** Correlation between the percentage of IFN-γ^+^ cells and the percentages of TNF-α^+^, NKG2D^+^, HLA-DR^+^, CCR7^+^, CD107a^+^ cells in NK cells, and correlation between the percentage of IFN-γ^+^ NK cells and the cytotoxicity of PBMCs. Each symbol represents an individual donor.

We also found that IFN-γ showed better correlation with the function of CD4^+^, CD8^+^ T cells, and NK cells than other markers. First, our results showed that the production of IFN-γ was significantly correlated with the production of other cytokines such as TNF-α and IL-2 in CD4^+^, CD8^+^ T cells, and NK cells (Figures 2A–C). Second, the production of IFN-γ was positively correlated with the expression of activation marker HLA-DR but was negatively correlated with the expression of chemokine receptor CCR7 (Figures 2A–C). CCR7 is involved in the migration of T cells to various secondary lymphoid organs, and its expression level is decreased along with the activation of T cells (22). Third, the production of IFN-γ in NK cells had a good correlation with their active receptor NKG2D (Figure 2C). Fourth, the production of IFN-γ was significantly correlated with both the expression of degranulation marker CD107a and the cytotoxicity to target cells in CD8^+^ T and NK cells (Figures 2B,C). These data demonstrate that IFN-γ production could be used as a symbol to reflect the function of CD4^+^, CD8^+^ T cells, and NK cells simultaneously.

### Determination of Standard Procedures for Whole Blood IFN-γ Secretion Assay

**Figure 3:**
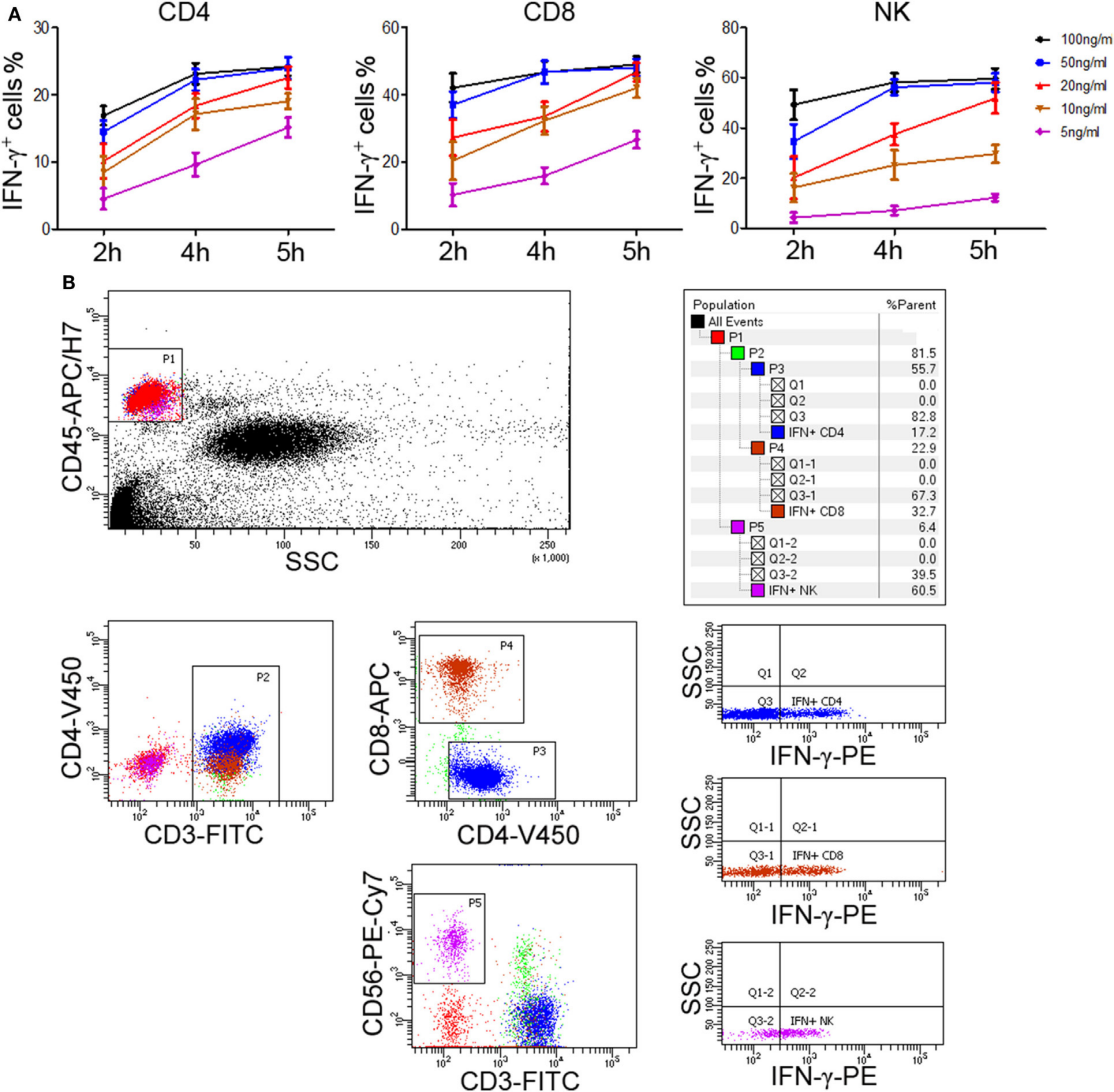
Determination of standard procedures for whole blood IFN-γ secretion assay. Diluted whole blood (1:5) was incubated with phorbol-12-myristate-13-acetate (PMA; 5, 10, 20, 50, 100 ng/ml), ionomycin (1 µM), and brefeldin A (1 µg/ml) for 2, 4, or 5 h, respectively. **(A)** The percentages of IFN-γ^+^ cells in CD4^+^, CD8^+^ T cells, and NK cells were evaluated by flow cytometry. Data are shown as mean ± SEM. **(B)** Diluted whole blood was stimulated with Leukocyte Activation Cocktail (BD GolgiPlug™, including 50 ng/ml PMA, 1 µM ionomycin, and 1 µg/ml brefeldin A) for 4 h. Representative flow cytometry gating strategy for identification of IFN-γ expression in CD4^+^, CD8^+^ T cells, and NK cells.

The above experiments used PBMCs to evaluate lymphocyte function. However, in order to improve the applicability in clinical practice, we decided to use whole blood instead of PBMCs to detect the secretion of IFN-γ. After comparing the effect of undiluted whole blood with diluted one, the diluted whole blood (1:5) showed better performance in IFN-γ secretion. We also compared the effect of different stimuli (including IL-2, IL-12, PHA, anti-CD3 plus anti-CD28, and PMA/ionomycin) on the secretion of IFN-γ by T cells and NK cells. Our results showed that only PMA/ionomycin could induce a large amount of IFN-γ secretion in CD4^+^, CD8^+^ T cells, and NK cells simultaneously (Figure S1 in Supplementary Material). Then, after trying different experimental conditions, we found that 50 ng/ml of PMA and 4 h of stimulation yielded a peak IFN-γ production in shortest time (Figure 3A). Furthermore, to detect the secretion of IFN-γ in CD4^+^, CD8^+^ T cells, and NK cells in one tube simultaneously, we used six fluorescent antibodies to label different lymphocyte subsets and the final results can be easily observed in our established flow cytometry analysis template (Figure 3B). Finally, we have established the standard procedures for evaluating the function of CD4^+^, CD8^+^ T cells, and NK cells based on IFN-γ secretion.

### Reproducibility

**Figure 4:**
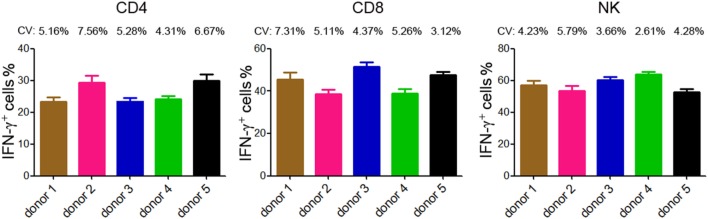
Reproducibility of intracellular IFN-γ detection by flow cytometry. Five healthy donors were recruited and three samples were taken from each individual (the time interval between two samples was 1 week). Intracellular staining of IFN-γ was performed in diluted whole blood after stimulated with Leukocyte Activation Cocktail (BD GolgiPlug™) for 4 h. The percentages of IFN-γ^+^ cells in CD4^+^, CD8^+^ T cells, and NK cells of each individual are shown in bar graphs. Data are shown as mean ± SD. CV, coefficient of variation.

The reproducibility is important for possible application of this method in clinical practice. We therefore tested the reproducibility of this lymphocyte function assay by repeating analysis of different samples obtained from five healthy individuals. Three samples were taken from each individual and the time interval between two samples was one week. The results of IFN-γ^+^ cells in CD4^+^, CD8^+^ T cells, and NK cells are shown in Figure 4. The coefficients of variation (CVs) were calculated by dividing the SD by the means and the CVs of each individual were below 10%. These data demonstrate that the reproducibility of this assay is acceptable.

### Establishment of the RIs of Lymphocyte Function

**Figure 5:**
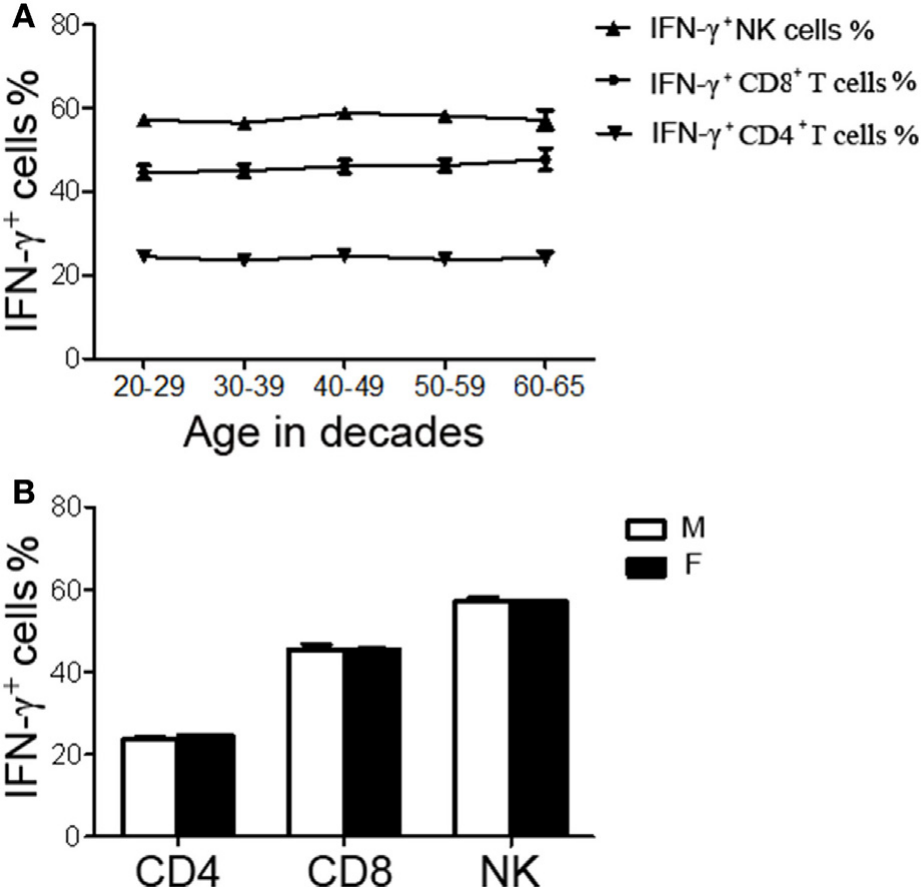
The production of IFN-γ^+^ cells in CD4^+^, CD8^+^ T cells, and NK cells in different age and gender groups. Diluted whole blood from 200 healthy adults was stimulated with Leukocyte Activation Cocktail (BD GolgiPlug™) for 4 h and analyzed by flow cytometry. **(A)** Liner diagrams showing the age-related changes in the production of IFN-γ in CD4^+^, CD8^+^ T cells, and NK cells. **(B)** Bar graphs showing the production of IFN-γ in CD4^+^, CD8^+^ T cells, and NK cells between different genders. Data are shown as mean ± SEM. M, male; F, female.

A total of 200 healthy adults (92 males and 108 females) aged 20–65 years were recruited in this part of study. The demographic and clinical characteristics of the participants are shown in Table 1. According to standard procedures described above, the production of IFN-γ in CD4^+^, CD8^+^ T cells, and NK cells in diluted whole blood was determined and shown in Table S1 in the Supplementary Material. We observed that the secretion of IFN-γ by CD4^+^, CD8^+^ T cells, and NK cells in different age and gender groups had no significant difference (Figures 5A,B). Furthermore, according to the production of IFN-γ in CD4^+^, CD8^+^ T cells, and NK cells, the RIs of lymphocyte function were calculated as the 2.5 percentile and the 97.5 percentile of the inferred normal distribution (Table 2). The mean and RIs of lymphocyte function were as follows: IFN-γ^+^ CD4^+^ T cells (%): 24.09 (15.31–34.98); IFN-γ^+^ CD8^+^ T cells (%): 45.55 (26.11–66.59); IFN-γ^+^ NK cells (%): 57.23 (39.43–70.79).

**Table 1:** Baseline characteristics of the healthy adults.

Characteristic	Value
Total	200
Male	92 (46%)
Female	108 (54%)
Age (years^a^)	37 (20–64)
BMI	22.36 ± 0.2005
<18.5	8 (4%)
18.5–24.99	174 (87%)
>25	18 (9%)
Systolic pressure	119.5 ± 1.255
Diastolic pressure	75.73 ± 0.9119
White blood cell (10^9^/L)	5.800 ± 0.0972
Neutrophil (10^9^/L)	3.385 ± 0.0886
Lymphocyte (10^9^/L)	1.915 ± 0.0365
ALT (U/L)	28.09 ± 0.7853
AST (U/L)	25.12 ± 0.6043
Glucose (mmol/L)	5.032 ± 0.1238

*^a^Presented as median and range*.

*Data were shown as number (%) or mean ± SEM*.

*BMI, body mass index; ALT, alanine aminotransferase; AST, aspertate aminotransferase*.

**Table 2:** Reference intervals of lymphocyte function.

Parameter	*n*	Mean ± SEM (%)	2.5th–97.5th percentile (%)
IFN-γ^+^CD4^+^ T cells	200	24.09 ± 0.3713	15.31–34.98
IFN-γ^+^CD8^+^ T cells	200	45.55 ± 0.7792	26.11–66.59
IFN-γ^+^ NK cells	200	57.23 ± 0.6247	39.43–70.79

### Validation of the RIs in Another Center

**Figure 6:**
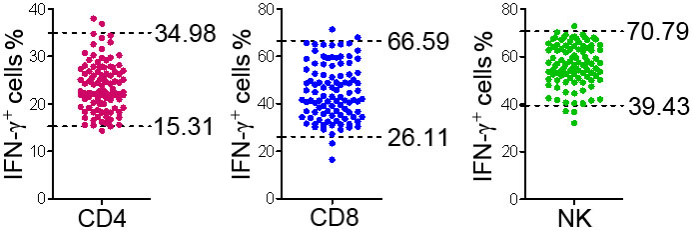
Validation of the reference intervals (RIs) of lymphocyte function in another center. Diluted whole blood from another 100 healthy adults recruited from another center was stimulated with Leukocyte Activation Cocktail (BD GolgiPlug™) for 4 h. The production of IFN-γ in CD4^+^, CD8^+^ T cells, and NK cells was measured by flow cytometry. Scatter plots showing the percentages of IFN-γ^+^ cells in CD4^+^, CD8^+^ T cells, and NK cells. The upper and lower dashed lines showing our established RIs of lymphocyte function.

To validate the established RIs of lymphocyte function, another 100 healthy adults (50 males, 50 females) were recruited from another center (Sino-French New City Branch of Tongji Hospital). The production of IFN-γ in CD4^+^, CD8^+^ T cells, and NK cells of these individuals was detected according to above procedures. For the function of CD4^+^ T cells, three individuals were out of RI; for the function of CD8^+^ T cells, four individuals were out of RI; for the function of NK cells, four individuals were out of RI (Figure 6). These data showed that greater than 95% of results were within the established RIs, which suggests that the established RIs of lymphocyte function are reliable and suitable for use in other centers.

### Validation of the RIs in Individuals with Different Immune Status

**Figure 7:**
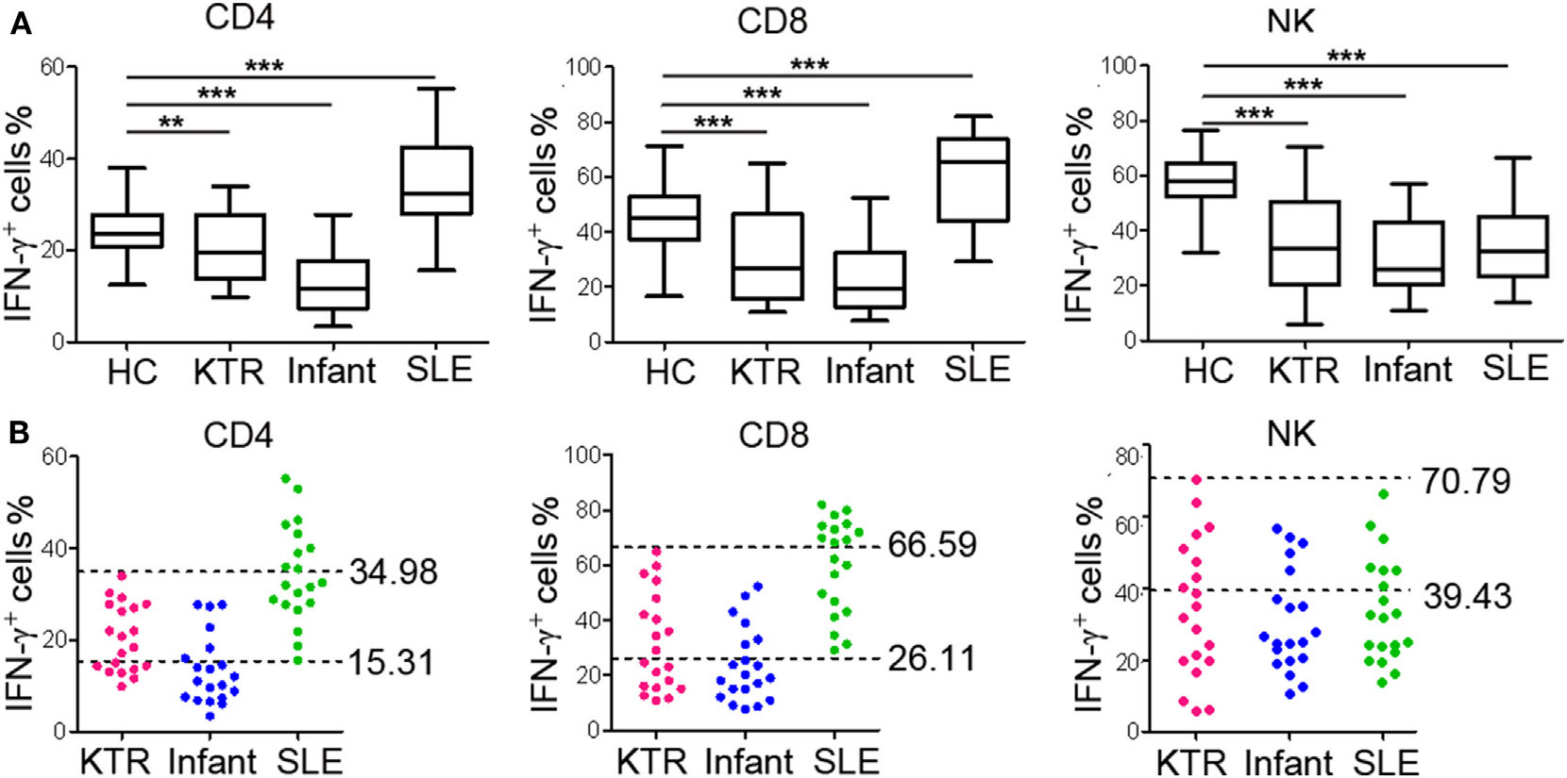
Validation of the reference intervals (RIs) in individuals with different immune status. Whole blood samples were collected from KTR (*n* = 20), infants (0–1 year, *n* = 20), and SLE patients (*n* = 20). Diluted whole blood was stimulated with Leukocyte Activation Cocktail (BD GolgiPlug™) for 4 h, and the production of IFN-γ was measured by flow cytometry. **(A)** The percentages of IFN-γ^+^ cells in CD4^+^, CD8^+^ T cells, and NK cells in healthy adults and individuals with different immune status are shown. The box plots depict the median, and 25th to 75th percentiles, and the whisker indicates the maximum and minimum values. **p < 0.01, ***p < 0.001. **(B)** Scatter plots showing the percentages of IFN-γ^+^ cells in CD4^+^, CD8^+^ T cells, and NK cells in individuals with different immune status. The upper and lower dashed lines showing our established RIs of lymphocyte function. HC, healthy controls; KTR, kidney transplant recipients; SLE, systemic lupus erythematosus.

To further validate the established RIs of lymphocyte function, we also recruited three groups of individuals with different immune status (20 KTR under immunosuppressive therapy; 20 infants aged 0–1 year; 20 newly diagnosed SLE patients before treatment). We observed that the function of CD4^+^, CD8^+^ T cells, and NK cells was all significantly decreased in KTR and infants compared with healthy adults. However, the function of CD4^+^ and CD8^+^ T cells was significantly increased and the function of NK cells was significantly decreased in SLE patients compared with healthy adults (Figure 7A). Furthermore, for the KTR, 40% (8/20), 50% (10/20), and 60% (12/20) of results were out of CD4^+^, CD8^+^ T cell, and NK cell function RIs, respectively; for the infants, 70% (14/20), 70% (14/20), and 75% (15/20) of results were out of CD4^+^, CD8^+^ T cell, and NK cell function RIs, respectively; for the SLE patients, 45% (9/20), 50% (10/20), and 65% (13/20) of results were out of CD4^+^, CD8^+^ T cell, and NK cell function RIs, respectively (Figure 7B). These data exhibit that the established RIs of lymphocyte function can be utilized to reflect the patients with different immune status.

## Discussion

Evaluation of lymphocyte function is an important tool in diagnosis, monitoring, and prognosis of immune-related diseases. Given that lymphocytes have various activities (e.g., activation, cytotoxicity, cytokine secretion), it is very complicated to evaluate different aspects of lymphocyte function simultaneously. The present methods such as CFSE proliferation assay and CD107a degranulation assay are complicated and have limitations in clinical practice (12, 13). Therefore, the methods which can be used to evaluate the function of different subsets of lymphocytes in a simple and rapid way are still needed. In this study, we found that IFN-γ production was correlated with the activation, cytotoxicity, and cytokine secretion of lymphocytes and could be used as a symbol of lymphocyte function. Thus, based on IFN-γ production, we can evaluate the function of CD4^+^, CD8^+^ T cells, and NK cells simultaneously using flow cytometry. Furthermore, we established the RIs of CD4^+^, CD8^+^ T cell, and NK cell function according to IFN-γ secretion assay, which could be used as an important tool in evaluating the immune status of hosts.

Cytokine monitoring has been proven useful in selection of immunosuppressive therapy, prediction of graft rejection, and progression of various diseases involving immunological abnormalities (23, 24). Previous studies have shown that IFN-γ production is a useful biomarker of graft dysfunction, cancer progression, and infectious disease prognosis (25–27). However, the choice of stimuli is an important issue for evaluation of IFN-γ production because different stimuli have specific mechanisms in activating lymphocytes. To determine the best one, we compared the effects of different stimuli including anti-CD3 plus anti-CD-28 (TCR/CD3 complex), PHA (mitogen receptor), IL-2 or IL-12 (cytokine receptor), and PMA/ionomycin (protein kinase C activator) on the secretion of IFN-γ by T cells and NK cells. Although IL-2, IL-12, PHA, anti-CD3 plus anti-CD28, and PMA/ionomycin can all activate lymphocytes, PMA/ionomycin is the best one to boost cytokine production with short-term stimulation (4–6 h). This is also in accordance with previous study indicating that PMA/ionomycin can rapidly induce the secretion of IFN-γ by directly activating protein kinase C as well as increasing intracellular calcium concentration (28). However, given that PMA/ionomycin stimulation bypasses surface receptor-mediated activation, the result of this stimulation may have some discrepancy with that of more physiological stimulus such as PHA. This is why PMA/ionomycin stimulation induces a different trend of IFN-γ secretion by CD4^+^, CD8^+^ T cells, and NK cells compared with PHA stimulation. Furthermore, previous studies have also shown that PMA/ionomycin stimulation can be used in evaluating the activity of lymphocytes, monitoring the immunosuppressive therapy, and predicting the outcome of graft rejection (9, 29, 30). Finally, we used PMA/ionomycin as stimulus to activate lymphocytes.

Various cytokines and receptors are changed in lymphocytes under different stimulation. We chose IFN-γ instead of other markers as a symbol of lymphocyte function according to the following reasons: (1) IL-2, IL-17, and IL-21 were mainly produced by CD4^+^ T cells and the production of TGF-β1 in both T cells and NK cells was low. Thus, these cytokines are not suitable to be used to evaluate the function of CD4^+^, CD8^+^ T cells, and NK cells simultaneously. (2) CCR7 was expressed on T cells but not on NK cells, while NKG2D was expressed on NK cells but not on T cells. (3) The expression of CD107a on CD8^+^ T cells and NK cells was relatively high, while its expression on CD4^+^ T cells was low. (4) Although IFN-γ and TNF-α could be detected in T cells and NK cells, the level of TNF-α was relatively lower than that of IFN-γ. Moreover, we have also analyzed the correlation of other cytokines such as TNF-α and IL-2 with lymphocyte activity and we found that IFN-γ has a better correlation with the function of CD4^+^, CD8^+^ T cells, and NK cells than these cytokines. Therefore, under the stimulation of PMA/ionomycin, the production of IFN-γ seems to be a prominent marker for evaluation of lymphocyte function.

Based on the secretion of IFN-γ and expression of degranulation marker CD107a, we have explored the RIs of NK cell function in our previous study (19). But actually, we found that the RIs of NK cell function established in our previous study are not suitable for use in clinical practice. First, this method needs to separate PBMCs by using density gradient centrifugation. This procedure is complicated and requires a lot of manpower. Second, this method needs to detect the expression of IFN-γ and CD107a after 24 h stimulation. This procedure is also complicated and will spend a lot of time. Third, this method can only determine the function of NK cells, whereas the function of other types of lymphocytes is also important for evaluation of the immune status of hosts. In order to improve the method, we have used the whole blood instead of PBMCs in a 4-h stimulation protocol in the present study. After labeling with six fluorescent antibodies in one tube, we can detect the function of CD4^+^, CD8^+^ T cells, and NK cells simultaneously. Finally, we successfully created a simple method to measure the function of lymphocytes according to IFN-γ secretion, and our data suggest that the RIs of lymphocyte function might have potential value in evaluating the abnormalities of patients in various clinical situations.

There are several advantages for using our established RIs to evaluate lymphocyte function. First, the method in the present study only requires 100 µl of whole blood for stimulation, which is acceptable in patients requiring periodic tests or having difficulty in collecting samples (e.g., severe patients, children, and old people). This method avoids the isolation of PBMCs from whole blood, which reduces the costs of manpower and resources. Furthermore, whole blood is the best matrix to analyze the biomarker, because the immune cells are maintained in an environment more similar to that found *in vivo*. Second, 4-h of PMA/ionomycin stimulation induces a great amount of IFN-γ secretion. This method is less-time consuming and the laboratory results can be reported within one day (within 8 h). Third, by multiple color flow cytometry, we can detect the production of IFN-γ in CD4^+^, CD8^+^ T cells, and NK cells simultaneously. Moreover, the flow cytometry has the advantages of high throughput and automation. Thus, this method is suitable for use in clinical laboratories. Finally, all the materials (fluorescent antibodies and leukocyte activation cocktail) used in the present study are commercially available, so this method can be readily standardized in different laboratories.

The following limitations of the present study should be mentioned. Previous studies have shown that lymphocytes exhibit phenotypically and functionally heterogeneous (31) and that age, sex, ethnicity, and environment of the studied populations can influence the RIs (32). The subjects enrolled in this study were obtained from Chinese Han nationality, so further investigation in various ethnic groups and multi-centers with a large sample size is required. In addition, this study only established the RIs of lymphocyte function in healthy adults. However, the understanding of lymphocyte function in children and older people is also very important and need special attention. Finally, because PMA/ionomycin stimulation bypasses TCR ligation, the function of lymphocytes detected under this stimulation might have some discrepancy with the real result of lymphocyte activity.

In conclusion, we developed a simple, rapid, accurate, and non-hazardous method to evaluate the function of CD4^+^, CD8^+^ T cells, and NK cells. We further established the RIs of the lymphocyte function in healthy adults. The established RIs play important roles in the evaluation of immune status of hosts and are found to have a broad application in different disease models, which suggests they are of important clinical value in the diagnosis, monitoring, and prognosis of immune-related diseases.

## Ethics Statement

This study was carried out in accordance with the recommentations of ethical committee of Tongji hospital, Tongji Medical College, Huazhong University of Science and Technology with written informed consent from all subjects. All subjects gave written informed constent with the Declaration of Helsinki. The protocol was approved by the ethical committee of Tongji hospital, Tongji Medical College, Huazhong University of Science and Technology.

## Author Contributions

HH, FW, and ZS designed experiments. HH, YZ, JY, LEM, MJB, and JW performed the experiments and analyzed the data. HH and FW wrote the manuscript. YL, LYM, and XW revised the manuscript. All authors read and approved the final manuscript.

## Conflict of Interest Statement

The authors declare that the research was conducted in the absence of any commercial or financial relationships that could be construed as a potential conflict of interest.

## Supplementary Material

Figure S1 Comparing the effect of different stimuli on the secretion of IFN-γ by CD4^+^, CD8^+^ T cells, and NK cells. Peripheral blood mononuclear cells (PBMCs) were isolated from whole blood of healthy adults (*n* = 10) and stimulated with IL-2, IL-12, PHA, anti-CD3 plus anti-CD28 for 24 h, respectively. The isolated PBMCs were also stimulated with phorbol-12-myristate-13-acetate/ionomycin for 4 h. After stimulation, the production of IFN-γ in CD4^+^, CD8^+^ T cells, and NK cells was detected by flow cytometry. **(A)** Representative FACS plots showing the expression of IFN-γ in CD4^+^, CD8^+^ T cells, and NK cells after different stimuli. **(B)** Bar graphs showing the percentages of IFN-γ^+^ cells in CD4^+^, CD8^+^ T cells, and NK cells under different stimuli, respectively. Data are shown as mean ± SEM.
