# Supplementary figures and images for: Establishment of the Reference Intervals of Lymphocyte Function in Healthy Adults Based on IFN-γ Secretion Assay upon Phorbol-12-Myristate-13-Acetate/Ionomycin Stimulation
Source: Front Immunol. 2018 Feb 7;9:172. doi: 10.3389/fimmu.2018.00172 (PMC5808316; doi:10.3389/fimmu.2018.00172)

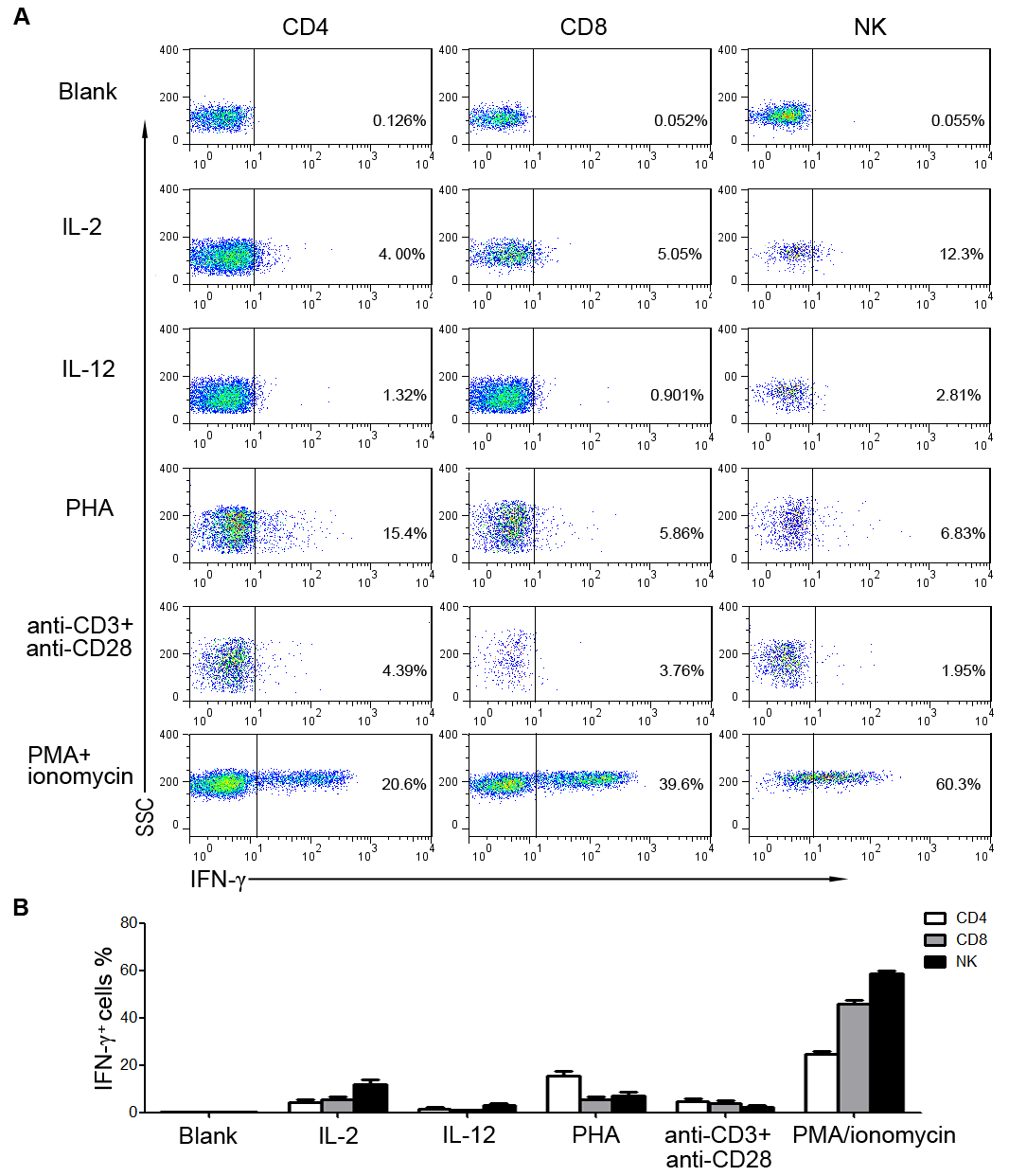

Supplement: Figure S1 — Comparing the effect of different stimuli on the secretion of IFN-γ by CD4+, CD8+ T cells, and NK cells. Peripheral blood mononuclear cells (PBMCs) were isolated from whole blood of healthy adults (n = 10) and stimulated with IL-2, IL-12, PHA, anti-CD3 plus anti-CD28 for 24 h, respectively. The isolated PBMCs were also stimulated with phorbol-12-myristate-13-acetate/ionomycin for 4 h. After stimulation, the production of IFN-γ in CD4+, CD8+ T cells, and NK cells was detected by flow cytometry. (A) Representative FACS plots showing the expression of IFN-γ in CD4+, CD8+ T cells, and NK cells after different stimuli. (B) Bar graphs showing the percentages of IFN-γ+ cells in CD4+, CD8+ T cells, and NK cells under different stimuli, respectively. Data are shown as mean ± SEM. [file image_1.tif]
