# Supplementary material for: Establishment of the Reference Intervals of Lymphocyte Function in Healthy Adults Based on IFN-γ Secretion Assay upon Phorbol-12-Myristate-13-Acetate/Ionomycin Stimulation
Source: Front Immunol. 2018 Feb 7;9:172. doi: 10.3389/fimmu.2018.00172 (PMC5808316; doi:10.3389/fimmu.2018.00172)
Supplement: Supplementary file 2 [file table_1.docx]

Supplement table 1 IFN-γ distribution in different gender and age groups

**IFN-γ^+^CD4^+^ T cells% IFN-γ^+^CD8^+^ T cells % IFN-γ^+^ NK cells %**

**Gender n**

Male 92 23.60±0.5444 45.56±1.136 57.32±0.8339

Female 108 24.50±0.5067 45.54±1.075 57.16±0.8930

**Age (years)**

20-29 44 24.53±0.8260 44.66±1.610 57.25±1.427

30-39 65 23.47±0.6110 45.02±1.590 56.45±1.230

40-49 46 24.75±0.7939 46.07±1.585 58.85±1.206

50-59 34 23.81±0.9506 46.29±1.528 58.23±1.411

60-65 11 24.05±1.5640 47.80±2.666 57.19±2.392

**Total** 200 24.09±0.3713 45.55±0.7792 57.23±0.6147

Data were shown as mean±SEM.
